# Appropriate Genetic Approaches for Heat Tolerance and Maintaining Good Productivity in Tropical Poultry Production: A Review

**DOI:** 10.3390/vetsci10100591

**Published:** 2023-09-25

**Authors:** Jiraporn Juiputta, Vibuntita Chankitisakul, Wuttigrai Boonkum

**Affiliations:** 1 Department of Animal Science, Faculty of Agriculture, Khon Kaen University, Khon Kaen 40002, Thailand; jiraporn.ju@kkumail.com (J.J.); vibuch@kku.ac.th (V.C.); 2 Network Center for Animal Breeding and Omics Research, Khon Kaen University, Khon Kaen 40002, Thailand

**Keywords:** heat stress, genetic method, sustainability, chicken

## Abstract

**Simple Summary:**

Tropical regions exhibit a wide range of climatic and environmental conditions. Therefore, to optimize the selection of genetic approaches to address heat stress affecting poultry productivity, this article compiles the different advantages and limitations of genetic methods for readers to have information to help them decide which method is suitable for their area and with genetic methods inevitably produces more sustainable results than other methods.

**Abstract:**

Heat stress is a major environmental threat to poultry production systems, especially in tropical areas. The effects of heat stress have been discovered in several areas, including reduced growth rate, reduced egg production, low feed efficiency, impaired immunological responses, changes in intestinal microflora, metabolic changes, and deterioration of meat quality. Although several methods have been used to address the heat stress problem, it persists. The answer to this problem can be remedied sustainably if genetic improvement approaches are available. Therefore, the purpose of this review article was to present the application of different approaches to genetic improvement in poultry in the hope that users will find suitable solutions for their poultry population and be able to plan future poultry breeding programs.

## 1. Introduction

The food shortage crisis is a significant concern in many countries [1,2,3]. The FAO [4] reported that the number of people affected by global food shortages has increased from 8.0% of the world population in 2019 to 9.8% in 2021, equivalent to 828 million people worldwide. Countries with a steady population growth rate, such as the Republic of Burundi, Yemen, Venezuela, and India, will be more affected than countries with declining or non-increasing rates [5]. In addition, the World Population Data Sheet [6] forecasted that the world population will increase by approximately 2.4% by 2050. As a result, food demand will increase accordingly. Meat is one of the foods that is experiencing an increasing demand for consumption. Poultry is the most consumed kind of meat, accounting for 43% of the world’s total meat consumption, followed by pork (33%), beef (19%), and lamb and goat (5%) [7]. In addition, the OECD-FAO [8] forecasted that by 2025, world meat production will likely increase by 16%, with poultry being the primary driver of overall meat production growth to support demand. However, one of the significant hurdles that could result in inaccurate forecasting of such output numbers is global climate change influencing heat stress effects. According to Copernicus [9], the global average temperature has increased by 2 °C compared to the database year 1850–1900; in particular, the environments in hot and humid tropical areas have a high risk of animals being affected by heat stress. Heat stress resulted in lower poultry productivity due to less feed intake, slower growth rate, and reduced fertility. It involves the immune system and body functions that change from routine [10]. Ultimately, if the bird cannot manage, severe heat stress can lead to mass deaths, which have widespread consequences, especially economic losses [11].

Several approaches have been used to reduce the severity of heat stress in poultry. The surrounding environment was modified using natural shading and increasing drinking water points on the farm [12]. A well-ventilated house with exhaust fans was designed to circulate air and insulation to maintain a stable indoor temperature [13,14]. Feed management techniques have been tried, such as scheduling feeding when environmental temperatures are lowest to reduce metabolic heat during hot weather [15,16,17]. Recent research highlights diverse dietary strategies to enhance chicken health and performance. Gouda et al. [18] advocate for L-ascorbic and folic acids, which improve antioxidant status and growth. Selenium nanoparticles, as noted by Lochi et al. [19], benefit body weight and intestinal health. According to Ogbuagu and Ayo [20], L-serine enhances meat quality and bone characteristics under heat stress. Du et al. [21] suggested that symbiotics can boost growth, immunity, and intestinal function, while Abbass and Abid [22] recommend using peppermint and fenugreek as natural additives to combat heat stress and improve productivity in chickens. Moreover, supplementation of vitamin E and organic compounds such as selenium and zinc (Se and Zn) in the diet increased the growth efficiency of chickens and reduced heat-related mortality [23,24,25,26]. Moreover, wet feeding was found to reduce thermal stress [27,28]. However, the methods mentioned above give good results only in the short term and do not solve the problem sustainably. In addition, some methods require a long time and a high investment budget, which may not be suitable for areas with limited investment budgets. For this reason, genetic approaches have been developed to breed poultry with increased heat tolerance while maintaining high productivity. These genetic advancements promise the future of poultry farming in hot and humid regions.

Therefore, we aimed to provide a clear understanding and guidelines for its appropriate application. This review article aims to research and collect data on the effects of heat stress on chickens and propose methods for breeding chickens appropriate to an area’s hot and humid conditions for future poultry genetic improvement.

## 2. Heat Stress in Poultry

**Figure 1:**
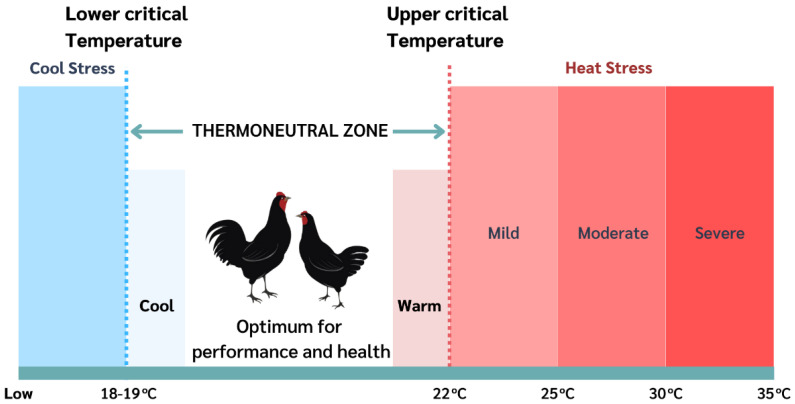
Different ambient temperature zones for poultry.

Heat stress is the body’s response to its inability to cope with the environment at high temperatures [29], which exceeds the animal’s ability to regulate temperature. This situation occurs due to the balance of the net heat energy transfer from the animal’s body to the surrounding environment and the amount of heat energy generated by the animal. As a result, the animal cannot adequately eliminate body heat [30,31]; consequently, the animal’s temperature rises, which results in heat stress. Heat stress is caused by changes in the animal’s surrounding environment, air temperature, relative humidity, solar radiation, wind speed, and metabolic rate of the animal’s body [32,33]. However, these effects harm the animal depending on the severity and duration of exposure to high temperatures [34,35]. Each species and age of poultry has an appropriate temperature range (thermoneutral zone), which can produce different results [36,37,38,39]. Moreover, it is the temperature range in which the animal has the lowest energy loss because it does not need to expend energy cooling or warming its body. In poultry, the optimum temperature for efficiency is 19–22 °C in layers [40] and 18–22 °C in broilers [41,42,43]. Temperatures above this range (upper critical temperature) will cause thermal stress. The stress levels can be divided into three levels: mild stress, 25 °C; moderate stress, 30 °C (there were signs of fatigue and lethargy) [12]; and severe stress, 35 °C [44]. At severe stress, chickens cannot dissipate body heat, resulting in organ function failure and death [45] (Figure 1). For example, Welay et al. [46] revealed that broilers exposed to temperatures above 32 °C exhibited diminished feed intake and body weight gain, indicating a direct temperature effect on broiler growth. Sohail et al. [47] revealed that broilers are subjected to chronic heat stress by reduced feed intake, lower body weight, and higher feed conversion ratio. In addition, several studies have shown impaired growth performance and meat in broilers subjected to heat stress [35,48]. For layers, Attia et al. [49] demonstrated detrimental effects on layers, such as reduced egg production, lighter eggs, and poorer eggshell quality. In native chickens, Boonkum et al. [50] found the effects of heat stress on reduced growth rate and genetic parameters at the temperature-humidity index of 76; meanwhile, Loengbudnark et al. [51] found genetic impacts of heat stress on egg production of Thai native chickens. 

## 3. Mechanism of Thermoregulation in Poultry

**Figure 2:**
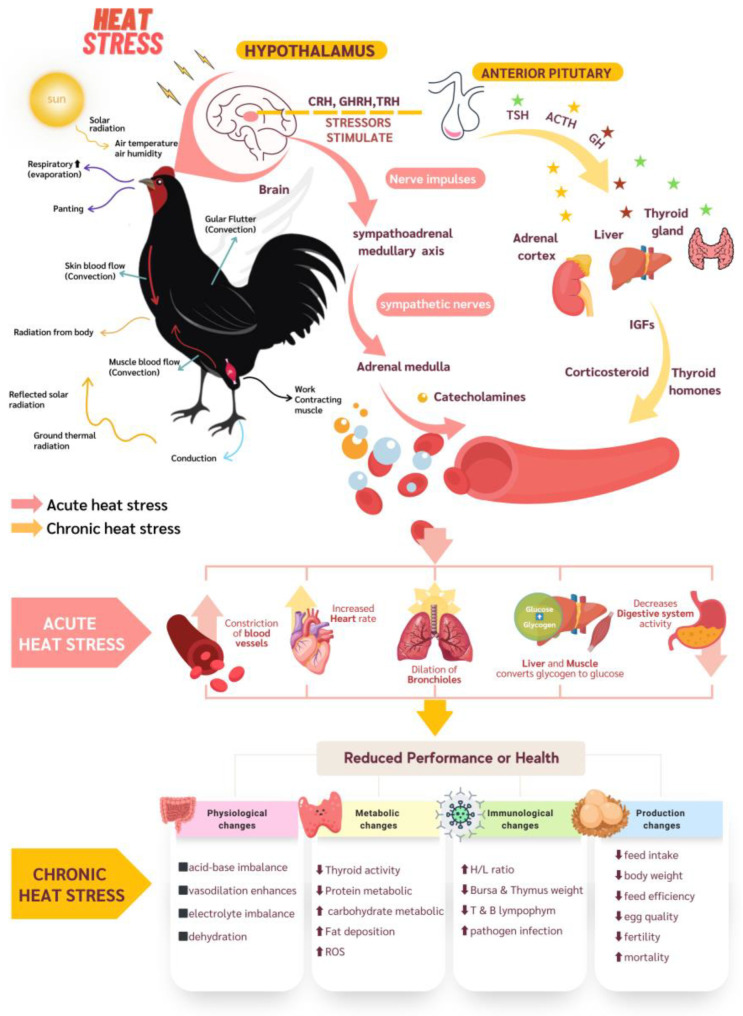
Heat stress response mechanism and effects in poultry.

Poultry is warm-blooded, meaning it can maintain a constant body temperature despite changes in ambient temperature [52]. Poultry begins to exhibit behavioral responses to expel excess heat, as shown in Figure 2. The cooling mechanism in poultry can be divided into four processes: (1) Conduction processes, for example, being in contact with the floor, planks, sidewalls, and cage floors inside the house [53,54,55] with a temperature lower than the animal’s body temperature, which causes internal body heat to flow along the surface. (2) Convection processes such as dilating blood vessels, observed from the darkening of body parts such as crest and wattle [56], will increase blood supply to the skin area. The skin is exposed to air and is easily carried away by wind. In addition, chickens can remove heat from the body by gular flutter [57] to allow more air to flow over the skin. (3) The evaporation process is an animal’s natural cooling mechanism based on the principle of latent heat transfer caused by the transition of water from liquid to gas to lower body temperature through a process known as panting. Heat can be exhausted from the body up to 40% of the total cooling capacity [58,59]. (4) Radiation is the heat transfer process from the chicken’s body to the environment through electromagnetic radiation [13], depending on the air around the chicken. The radiating heat capacity is also lower if the ambient temperature is high. However, if the ambient temperature is lower than the chicken’s body temperature, the radiating heat capacity is greater [60]. If the ambient temperature is above 40 °C, the chicken cannot radiate out of the body at all. Radiant cooling accounts for approximately 5% of total cooling [61].

The mechanisms for the heat stress response of the nervous and endocrine systems are shown in Figure 2. The amount of time a bird spends in high temperatures can be classified into acute and chronic. Acute stress is distinct for short-lived and frequently developing in response to acute and temporally limited stress-inducing stimuli. Meanwhile, chronic stress is characterized by protracted and enduring stress conditions that last longer. Consequently, there exists a distinct variation in the hormonal response, which can be delineated as follows: (1) Acute heat stress is characterized by a swift and immediate response aimed at priming the individual for the “fight or flight” reaction [62]. The animal is at risk due to the interplay of its neurological systems and hormonal responses. The phenomenon of stress triggers the activation of the sympathetic nervous system stimulation secretion. Catecholamines (adrenaline and noradrenaline) are secreted from the adrenal medulla. Both adrenaline and noradrenaline lead to the mobilization of various glucose sources, which are promptly released in substantial amounts as a source of energy during a response to heat stress. This mobilization involves stimulating an increased heart rate, constricting blood vessels in the viscera and skin, dilating blood vessels in the heart and skeletal muscles, converting glycogen into glucose in the liver, and dilating the bronchial tubes. These two hormones also inhibit other energy-demanding bodily processes, including digestion, growth, immune, and reproductive functions [63]. (2) Chronic heat stress, the hypothalamic-pituitary-adrenal system (HPA) synthesizes corticotropin-releasing hormone (CRH), growth hormone-releasing hormone (GHRH), and hydrotropic-releasing hormone (TRH). These hormones are then released into the anterior pituitary gland to stimulate the synthesis of adrenocorticotropic hormone (ACTH), growth hormone (GH), and thyroid stimulating hormone (TSH), respectively, which is the hormone that stimulates the production of corticosterone in the adrenal cortex gland, IGFs in the liver, and thyroid hormones (triiodothyronine (T3) and thyroxine (T4)) in the thyroid gland. Corticosterone, insulin-like growth factors (IGFs), and thyroid hormones help make glucocorticoids, widen blood vessels, break down fat and proteins, and reduce inflammation [64]. The primary hormone in the glucocorticoid group, corticosterone, stimulates hepatic gluconeogenesis by increasing the production of enzymes involved in converting the amino acids glycerol and lactate into glucose [65]. It is essential in regulating glucose, protein, and lipid metabolism, suppressing the immune response, and helping maintain blood pressure [66]. The increase in ACTH and glucocorticoid (GC) levels also inhibits other energy-consuming processes in the body, such as growth, immunity, reproduction, and digestion [67]. GC hormone can reduce protein synthesis and decrease cell lipolysis levels, increasing fat accumulation [68]. Therefore, chickens exposed to prolonged stress may develop muscle weakness and reduced antibody production and response, impairing immune function. Animals experiencing thermal stress are also exposed to higher levels of reactive oxygen species (ROS), which can cause lipid peroxidation and oxidative protein changes [35]. These are major causes of cellular DNA damage, fats, proteins, carbohydrates, and other molecules [69,70], called heat shock [71].

## 4. Effects of Heat Stress on Poultry

Heat stress negatively impacts the global poultry industry, as thermoregulatory mechanisms cause heat stress effects. The effects of heat stress on physiological, metabolic, immunological, and productivity changes in poultry are presented in Table 1, and details are shown below.

### 4.1. Physiological Changes

Under heat stress conditions, birds utilize various methods for decreasing body temperature, including panting, to dissipate excess heat. By panting, birds increase the airflow over their respiratory surfaces, promoting evaporative cooling through the moist lining of the respiratory tract [72]. However, it is noted that rapid and shallow breathing leads to the loss of carbon dioxide and, subsequently, the imbalance of plasma bicarbonate [71]. The disturbance of acid–base balance affects eggshell mineralization by decreasing plasma-free calcium levels. Finally, it adversely affects laying hens by producing eggs with thin shells, misshapen eggs, or eggs with lower internal quality [73]. Heat stress can lead to alterations in blood circulation patterns in poultry [62]. Vasodilation enhances heat dissipation by transferring heat from the body’s core to the skin surface, promoting radiant and convective heat loss [74]. This redirection of blood can reduce blood flow to other organs and may impact the overall physiological functioning of various organ systems [75]. Heat stress can disrupt the electrolyte balance in poultry [76]. Birds lose essential electrolytes, such as sodium, potassium, and chloride, through increased panting. These electrolytes are crucial for maintaining proper cellular function, and imbalances can lead to physiological disturbances and potential health issues [77]. Under heat-stress conditions, birds might be dehydrated if adequate water is not available for rehydration [78]. Therefore, an increased demand for water consumption is required for thermoregulation and osmoregulation [79].

### 4.2. Metabolic Changes

Heat stress triggers hormonal responses in poultry, such as thyroid and GC. Thyroid hormone plays a crucial role in regulating the rate of protein breakdown and responses to maintain the body temperature. During heat stress conditions, the decrease of plasma T3 and T4 concentrations was observed to decrease metabolic heat production [80]. Meanwhile, releasing GC hormones, such as corticosterone, during heat stress conditions resulted in increased fat accumulation [81]. Therefore, the heat stress conditions lead to decreased and increased protein content and fat deposition in chicken meat muscles, declining meat quality [82]. Besides meat quality, the synthesis of proteins involved in growth, reproduction, and immune function may be reduced, leading to slower growth rates, compromised reproductive performance, and decreased immune response. Furthermore, heat stress conditions induced oxidative stress in poultry [83]. ROS production exceeds the bird’s antioxidant defense capacity, leading to oxidative damage to cells and tissues [84,85].

### 4.3. Immunological Changes

Heat stress significantly impacts the immune function of poultry birds, leading to changes in the proportion of leucocytes in the blood and an increase in the heterophil: lymphocyte (H/L) ratio [86,87]. Heterophils are essential for innate immunity and phagocytosis, acting as the first line of defense after infection. The H/L ratio is a widely used indicator of HS, with increasing numbers found in the blood during the initial phase of the inflammatory response [88,89]. Heat stress also decreases the weight of lymphoid organs and the thymus [90,91], reducing T and B lymphocytes and decreasing antibody production [92]. Mashay et al. [73] claimed that heat stress exposure reduced the number and activities of leukocytes, as indicated by lower total white blood cell count in the heat-stressed group. This reduction may be due to inflammatory cytokine production, which stimulates corticosterone production from the adrenal gland. Therefore, the incidence of diseases caused by different pathogens increased in birds under heat stress conditions [93,94,95]. The decrease in immunity and more susceptibility to pathogens suggest a link between the nervous system and the immune system [94,96,97]. It inferred that heat stress also suppresses immune function and impairs production performance.

### 4.4. Productivity Changes

Heat stress can have significant adverse effects on the productivity of poultry. When poultry experiences heat stress, birds often exhibit reduced feed intake, a phenomenon known as anorexia. The decreased appetite is a natural response to reduced metabolic heat production during digestion [98,99]. However, reduced feed intake can lead to inadequate nutrient intake, slower growth rates, and reduced weight gain [100], resulting in compromised performance and overall productivity [101]. This can have economic implications for poultry farmers. Furthermore, heat stress negatively impacts the feed conversion efficiency of poultry [102]. Feed conversion efficiency refers to the ability of birds to convert feed into body weight. In addition, Nanto-Hara et al. [103] reported that heat stress also results in intestinal damage, making it easier for bacteria to enter the circulatory system, causing infection [104], which would affect nutrient absorption of an already reduced volume of feed intake. In laying hens, it can lead to decreased egg production, reduced egg size, and poor egg quality [40,98,105]. Heat-stressed hens may produce smaller eggs with thin shells, misshapen eggs, or eggs with lower internal quality [73]. Additionally, heat stress can disrupt the egg-laying cycle, leading to irregular or decreased egg production. Additionally, heat stress can negatively affect the fertility of male and female breeding poultry [106]. High temperatures can impair sperm production and reduce sperm quality in males, decreasing fertility rates [107]. Heat stress in females can result in poor egg fertilization, decreased embryo development, and reduced hatchability rates [108]. Prolonged exposure to high temperatures can lead to increased mortality rates in poultry. Heat stress weakens birds, making them more susceptible to diseases, infections, and other health issues [77,109]. Mortality rates may rise due to heat-related complications, such as heat exhaustion, heat stroke, or increased disease susceptibility [109].

**Table 1:** The effects of heat stress on physiological changes, immune system, and productivity changes in poultry.

Parameters	Effects of Heat Stress	References
**Physiological changes**		
Acid–base imbalance	respiratory alkalosis can occur when the body’s pH is shifted towards alkalinity due to a reduction in blood carbon dioxide (CO_2_) levels.	Popoola et al. [71]
Vasodilation	increases skin-surface blood vessel dilatation. this enhances radiative and convective heat loss from the core to the skin.	Chaiyabutr et al. [62]; Mota-Rojas et al. [74]; Hall et al. [75]
Electrolyte Imbalance	sweating and pant during heat stress, losing sodium chloride, potassium, and chloride.	Nawab et al. [76]; Wasti et al. [77]
Dehydration	rapid respiration risks dehydration and electrolyte imbalances due to higher water loss.	Khan et al. [78]
**Metabolic changes**		
Thyroid activity declines	diminished thyroid hormone levels can diminish poultry metabolic rates, affecting growth and performance.	Del Vesco et al. [80]
Decreases Protein Metabolism	growth, reproduction, and immunity may be affected by decreased protein synthesis.	Zaboli et al. [82]
Increased Carbohydrate Metabolism	heat stress can elevate blood glucose levels through stress hormone release, potentially causing hyperglycemia.	Kikusato and Toyomizu [81]
The accumulation of fat increases	subcutaneous fat may decrease and abdominal fat rise. high temperatures reduce adipose tissue lipogenesis, altering meat quality and egg yolk composition.	Zaboli et al. [82]
Increased ROS	ROS from oxidative stress exceeds the bird’s antioxidant defenses. this damages tissues and cells.	Song et al. [84] Nanto-Hara et al. [85]
**Immune changes**		
A higher H/L ratio.	heterophil to lymphocyte (h/l) ratios rise during heat stress, indicating immune system alterations.	Soleimani et al. [88]; Al-Murrani et al. [89]
Bursa and thymus weight decline.	prolonged heat stress can reduce bursa and thymus weights, affecting lymphoid organ growth and function.	Hirakawa et al. [85]; Kammon et al. [91]
Reduced T and B lymphocyte activity.	heat stress reduces t and b lymphocyte function, lowering the immune system’s ability to fight infections.	Honda et al. [92]; Mashaly et al. [73]
Pathogen susceptibility rises.	heat stress can decrease poultry immune systems, making them more susceptible to diseases.	Alhenaky et al. [93]; Quinteiro-Filho et al. [94]; Ahmad et al. [95]
**Productivity changes**		
Reduced Feed Intake	lead to decreased appetite in poultry, resulting in lower feed consumption.	Rowland et al. [98]; Mazzoni et al. [99]
Reduced body weight	exposed to heat stress may experience slower growth rates and reduced body weight gain.	Awad et al. [100]
Feed efficiency reduction	The impairment of feed conversion efficiency results in elevated feed costs.	Sohao et al. [47]
Egg production decline	laying fewer eggs of reduced size and quality.	Yan et al. [40]; Loengbudnark et al. [51]; Rowland et al. [98]
Reducing fertility	impair the fertility of breeding poultry, leading to decreased hatchability.	Donoghue et al. [105]; Olusegun and Alabi [106]
Mortality rises	mortality rates can rise due to heat stress-induced physiological strain.	Aguanta et al. [109]

## 5. Genetic Approaches to Address Heat Stress in Poultry

**Figure 3:**
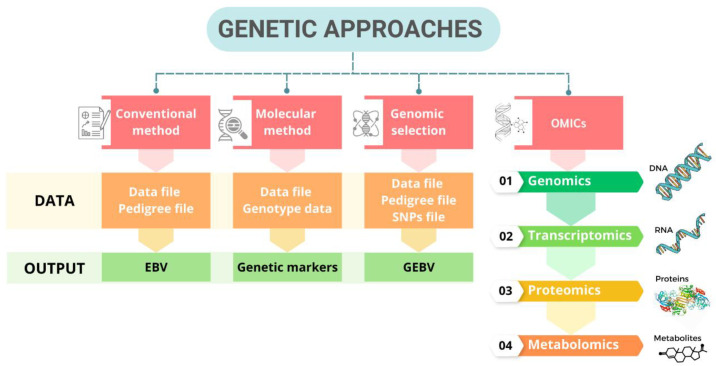
Genetic approaches to address heat stress in poultry.

Presently, there are four genetic approaches to reducing heat stress to improve poultry productivity: (1) the traditional method (EBVs), (2) the use of marker-assisted selection (MAS), (3) genomic selection, and (4) OMICS technology (Figure 3). Hereinafter, we will discuss each method in detail, including comparing differences in different dimensions and suggestions that may help in future decision making.

### 5.1. Conventional Method

Mating between high-yielding chicken breeds and native chicken breeds for the benefit of environmental adaptation and heat tolerance is a method that is widely used in many countries [110,111], but it may make purebred chickens more productive. The number of purebred chickens is steadily decreasing, which may not be suitable for animal genetic stability. At the same time, the temperature and humidity, which are calculated as the temperature–humidity index (THI), are used to assess the heat stress threshold for which performance and production begin to decline and to regress phenotypic performance on the THI value to quantify the genetic parameters of thermotolerance. The identification and selection of heat-tolerant animals is an important strategy for minimizing the effects of heat stress on dairy cattle productivity [112,113]. Thus, it is crucial to include heat adaptive parameters in the selection objective of dairy cow populations. Traditional models for describing an animal’s production performance in response to increased heat stress, known as the broken line (BL) model, assume that production does not change in the thermoneutral zone, and after the threshold point, production decreases linearly [114]. An alternative is to model the animal’s productive response using a reaction norm that uses polynomials. This approach offers higher flexibility than the BL [115]. In poultry, THI that exceeds a particular threshold for poultry often leads to significant declines in feed intake and body weight, lowered fertility, and a significant increase in mortality rate and physiological response. Boonkum et al. [50] estimated the impact of heat stress on the genetic absolute growth rate (AGR) in Thai native chickens and Thai synthetic chickens (chickens that have undergone genetic enhancement through the crossbreeding of commercial chickens with indigenous chicken breeds) and found a THI of 76. Compared to that of native Thai chickens, Thai synthetic chickens’ growth rate decreased more dramatically. Additionally, Loengbudnark et al. [51] investigated the effects of heat stress on the genetics of monthly eggs and found that monthly egg production started to decrease when the THI was 74.

### 5.2. Molecular Method by Marker-Assisted Selection

Marker-assisted selection (MAS) is a breeding technique used in agriculture and genetics to improve the efficiency and precision of selecting desirable traits in plants, animals, and other organisms [116]. MAS combines traditional breeding methods with molecular markers, which are specific sequences of DNA that can be easily detected and associated with particular traits or characteristics. By utilizing MAS in selecting heat tolerance genes in poultry, breeders can more effectively and efficiently identify individuals with desirable heat tolerance traits [117,118]. This targeted selection based on genetic markers enables breeders to improve heat tolerance in poultry populations more rapidly than relying solely on phenotypic evaluations [119]. Ultimately, this can contribute to developing poultry breeds better adapted to withstand high-temperature environments, resulting in improved productivity, health, and welfare in heat-stressed conditions.

At present, many genes are involved with heat stress effects (Table 2), which can be divided into two types: (1) directly controlled genes, namely, the heat shock factor (HSF) and heat shock protein (HSP) gene families; these work together to help reduce protein folding and are involved in important cellular defense mechanisms during exposure to hot environments [120]. Most of the major regulatory genes, HSF1 and HSF3, are involved in the regulation of HSPs; HSP27, HSP60, HSP70, and HSP90 are classified according to their molecular weights [121]. Cedraz et al. [120] found that HSP70 expression in commercial broilers is higher than that in native broilers during heat stress, particularly in the expression of the HSF1 and HSF3 genes. Furthermore, Duangjinda et al. [122] investigated the influence of the HSP70 genotype on heat tolerance in native chickens and discovered that the HSP70 genotypes displayed various tolerances to heat stress. It was discovered that the C2C2 genotype is susceptible to heat stress. As a result, commercial poultry breeding programs may choose to use C1C1 or C1C2 genotypes to improve heat tolerance. (2) Indirectly regulated genes from previous studies found that the HSF and HPS genes play major roles in regulating the heat response, and other genes that play a role in the regulation of apoptosis (RB1CC1, BAG3) [116,123], energy uptake and metabolism (GLUT-2, FABP1, CD36, FGA, LOXL2, GINS1, RRM2) [124,125], and immune response (HS3ST5, NFAT5, PDK) [12,126,127,128]. Several candidate genes involved in arid adaptation play a role in the heat stress response, as shown in Table 2. Nevertheless, some genes have been identified, their function in relation to heat stress has not yet been determined, such as CEP78, MEF2C, VPS13A, and ARRDC3, which may play an important role in regulating heat stress in poultry [129].

### 5.3. Genomic Selection

Genomic selection (GS) is a breeding approach that uses genomic information to predict the genetic merit of individuals for specific traits [137]. It is a form of marker-assisted selection that leverages high-density genotyping or whole-genome sequencing data to estimate the breeding value of individuals based on their genomic profiles. This approach has gained popularity in plant and animal breeding because it enables a more accurate and efficient selection of desirable traits [138,139]. Genomic selection has revolutionized breeding programs in various plant and animal species, leading to accelerated genetic progress, more efficient use of resources, and the development of improved varieties or breeds with desired traits [130,140]. In the context of genomic selection (GS) [141], there are generally two main types or approaches (Table 3). (1) Marker-based genomic selection relies on the analysis of genetic markers, such as single nucleotide polymorphisms (SNPs), to predict the genetic merit of individuals. The markers are genotyped or sequenced across the genome, and statistical models are developed to estimate the genomic estimated breeding values (GEBVs) based on the marker profiles. These GEBVs are used for selection decisions, and individuals with higher GEBVs are preferred as parents for the next breeding cycle. (2) In whole-genome selection, also known as genomic prediction, the entire genome of individuals is analyzed rather than specific genetic markers. This approach involves high-density genotyping arrays or whole-genome sequencing to obtain comprehensive genetic information. Statistical models, such as genomic best linear unbiased prediction (GBLUP) or Bayesian methods, are applied to estimate the genomic breeding values. The models capture the collective effects of numerous genetic markers distributed throughout the genome, allowing for the prediction of breeding values based on complete genomic information. Several studies have investigated the application of genomic selection to improve heat tolerance in chickens. For instance, researchers have used high-density genotyping arrays to identify genetic markers associated with heat tolerance traits and then applied genomic prediction models to estimate breeding values for heat tolerance. This approach enables the selection of individuals with higher genetic potential for heat tolerance, leading to more heat-resistant chicken lines [142]. In addition, Bjorkquist et al. [143] conducted studies to select improved adaptability to high temperatures in broiler chickens, which are bred for meat production. By combining genotyping data with phenotypic records of broilers reared under heat stress conditions, genomic selection models have been used to predict breeding values for heat tolerance. This allows breeders to identify and select broilers with enhanced thermotolerance traits, improving performance in high-temperature environments. Genomic selection has also been explored to improve the thermotolerance of laying hens raised for egg production [144]. By genotyping laying hens and employing genomic prediction models, breeders can predict the genetic merit of individuals for heat tolerance traits.

### 5.4. OMICS Technology

Several omics studies have been conducted in poultry to investigate the molecular mechanisms and genetic responses associated with heat stress (Figure 3). These studies have utilized genomics, transcriptomics, proteomics, metabolomics, and other omics approaches to gain insights into the physiological and molecular changes that occur in response to heat stress in poultry (Table 4). Genomic studies have examined the genetic variations and genomic regions associated with heat tolerance in poultry. These studies have involved genome-wide association studies (GWASs) or genomic selection approaches to identify candidate genes or genetic markers associated with heat tolerance traits [149]. Transcriptomic analyses have been used to investigate gene expression changes in response to heat stress in poultry. These studies have identified heat stress-responsive genes, pathways, and regulatory networks involved in thermoregulation, immune response, oxidative stress, metabolism, and other relevant processes. Transcriptomic data have been generated using microarray technology or next-generation sequencing techniques, such as RNA sequencing (RNA-seq) [150]. Proteomic studies have examined the changes in protein expression, posttranslational modifications, and protein–protein interactions in response to heat stress in poultry. These studies have identified heat shock proteins, antioxidant enzymes, and other proteins involved in stress responses and cellular defense mechanisms. Mass spectrometry-based proteomics approaches have been employed to profile protein expression patterns under heat stress conditions [151]. Metabolomic analyses have been used to investigate the metabolic changes and alterations in metabolite profiles associated with heat stress in poultry. Metabolomics studies have revealed changes in energy metabolism, amino acid metabolism, lipid metabolism, and other metabolic pathways in response to heat stress. Metabolites have been analyzed using liquid chromatography–mass spectrometry technique (LC–MS) [142]. These omics studies have provided valuable insights into poultry’s molecular and physiological responses to heat stress. They have helped identify potential biomarkers, pathways, and genetic markers associated with heat tolerance, which can inform breeding programs aimed at developing more thermotolerant poultry breeds and improving management strategies to mitigate the negative impacts of heat stress in poultry production.

## 6. Challenges of Improving Poultry Genetics in Tropical Areas

Genetically improving tropical poultry presents several challenges due to the specific environmental conditions and genetic traits of the birds. Some of the key challenges include the following: (1) Heat stress can negatively impact bird growth, feed conversion efficiency, and overall health. Therefore, one of the primary challenges is to develop poultry strains or breeds that are better adapted to hot and humid climates. (2) Genetic improvement programs aim to develop poultry breeds that are efficient in converting feed into body weight or egg production. This includes selecting birds with traits such as improved feed conversion ratios, increased feed intake to get better growth, and enhanced nutrient utilization. (3) High temperatures and humidity can adversely affect poultry reproductive performance, including reduced fertility rates and increased embryonic mortality. Genetic improvement efforts need to address these challenges by selecting birds with better reproductive traits, including improved egg production, hatchability, and fertility under tropical conditions. (4) Selective breeding programs need to consider the optimum body size and growth rate for tropical poultry that align with the local market demands. This may involve striking a balance between fast growth and efficient resource utilization, taking into account factors such as heat stress and feed availability. In addition, tropical regions exhibit a wide range of climatic and environmental conditions. Genetic improvement programs should consider the specific needs and challenges of different geographical regions within the tropics. Developing locally adapted poultry strains can help optimize performance and productivity under varying environmental conditions. Finally, ensuring genetic diversity is crucial for the long-term sustainability and adaptability of tropical poultry populations. Genetic improvement programs must carefully manage and maintain diverse genetic resources to prevent inbreeding depression and preserve the resilience of bird populations. Addressing these challenges requires a comprehensive approach that combines traditional breeding techniques, molecular genetics, and genomic selection methods, and we have compared the differences in using each method in Table 5 for readers to consider and use appropriately. In the end, collaboration between poultry breeders, geneticists, and researchers is essential to overcome these obstacles and achieve significant genetic improvements in tropical poultry.

## 7. Conclusions

The effects of climate change on poultry production are significant, particularly in terms of heat stress in birds. Although genetic improvement may not be the only method to reduce heat stress in poultry, it is unquestionably one that will produce long-term results and provide animals with the ability to improve their genetics in order to adapt to more severe weather in the future. The fact that each generation inherits the DNA of the previous generation is a crucial reason why thermophilic breeding is essential for the advancement of the global poultry production system. To address the challenge of sustaining a growing global population in a sustainable manner, particularly in regions with limited resources, it is essential to combine this strategy with practical, immediate measures. A holistic approach that incorporates multiple strategies will be the most effective way to ensure food security in the face of climate change and intensifying heat stress.

## Figures and Tables

**Table 2:** Gene markers that play a role in the response to heat stress in poultry.

Genes	Expression	Heat Control Functions	References
HSPA2, HSPH1, HSP25	Increase	provide cellular protection and healing.	Wang et al. [116]
RB1CC1, BAG3, CITED2	Increase	negative regulation of apoptosis and programmed cell death.	Wang et al. [116]; Luo et al. [123]
ID1	Decrease	It plays a role in embryonic development, tissue regeneration, and the control of cell proliferation.	Luo et al. [123]
HSP90B1, HSPD1, PDIA2, HSPA5	Increase	stabilize and refold denatured proteins in the endoplasmic reticulum and mitochondrial.	De Maio and Vazquez [130]
HSF1, HSF3	Increase	protects cells from heat damage.	Cedraz et al. [120]; De Maio and Vazquez [130]
HSP70, HSP90, HSP40	Increase	stabilize and refold denatured proteins, which is crucial for heat-stress cell survival.	
SERPINH1	Increase	facilitate protein folding, reduce aggregation, and recover misfolded proteins.	Wang et al. [125]; De Maio and Vazquez [130]
GLUT-2, FABP1, CD36	Decrease	decrease feed intake and intestinal damage.	Sun et al. [124]
TRMT1L	Increase	require for redox homeostasis to ensure proper cellular proliferation and oxidative stress survival.	Dewe et al. [131]; Walugembe et al. [132]
HS3ST5	Unknown	involve immunity and defense molecular functions.	Walugembe et al. [132]; Szauter et al. [133]
EOMES	Increase	stimulate immunity and control homeostasis.	Walugembe et al. [132]; Zhang et al. [134]
NFAT5, NF-κB	Increase	stimulate the expression of various proinflammatory cytokines.	Tellechea et al. [126]; Zhang et al. [134]
MRPL42	Increase	disrupt of DNA synthesis, transcription, RNA processing, and translation.	Van Goor et al. [117]
EDN1	Unknown	augment apoptosis in cancer cells induced by mild hyperthermia.	Wang et al. [116]
ACSF	Unknown	alter in energy metabolism during heat stress.	Tian et al. [135]
CYP4V2	Increase	increase fat deposition.	Claire De’Andre et al. [136]
PLCB4	Increase	assist in the regulation of metabolic energy	Nanaei et al. [118]
H1F0, ACYP	Increase	reduce heat-induced apoptosis and repair DNA damage.	Srikanth et al. [127]
PDK	Increase	maintain glucose and reduce heat from combustion.	Luo et al. [123]; Kumar et al. [128]

**Table 3:** Using GWAS and SNP to characterize heat resistance in poultry.

Number of SNPs	The Number of the Genotype	Breeds	Traits	References
23,098 SNPs	192	Taiwan indigenous chickens	Pathways associated with thermotolerance	Zhuang et al. [144]
580,954 SNPs	200	Taiwan country chickens	Body temperature change	Zhuang et al. [145]
113,344 SNPs	118	White Leghorn layer line.	Mortality in a white egg layer line	Wolc et al. [146]
304,500 SNPs	526	Hy-Line Brown	Controlling traits related to NDV infection during heat stress	Saelao et al. [147]
56,702 SNPs	206	Scaleless chickens	Feather development	Wells et al. [148]
210,117 SNPs	458	broiler × Fayoumi	Body temperature, body weight, breast yield, and digestibility	Van Goor et al. [117]
261,509 SNPs	374	White Leghorns	Production traits, feed intake, body weight, digestibility, egg quality	Rowland et al. [98]

**Table 4:** A study of OMICS technologies related to heat stress in poultry.

Techniques	Chicken Breeds	Analyzed	Genes	Functions	References
Genomics	Native Chickens	Blood and Muscle	BVES, SMYD1, IL18, PDGFRA, NRP1, CORIN	The circulatory system and blood vessel development	Gu et al. [152]
			SIM2, NALCN	Central nervous system development	
			CLPTM1L, APP, CRADD, PARK2	Related to apoptosis	
			AHR, ESRRG, FAS, UBE4B	Responded to stimuli	
			FABP1	Fatty acid metabolism	
	Fayoumis	Blood	MAP3K3, SOCS2	Cellular response to stress suppressing cytokine signaling.	Van Goor et al. [117]
		Blood	MAPKBP1, SPON1	Response to heat stress	Asadollahi et al. [153]
	Taiwan country chickens	Blood	CTL, H4R0, H4R2, H4R6	Response to acute heat stress	Cheng et al. [154]
	Native Chickens	Blood	SLC33A1, TSHR, NDUFS4	Biomarkers to assess the adaptation to extreme environments.	Shi et al. [155]
	Hy-Line Brown	Blood	CAMK1d, CCDC3 TIRAP, ETS1, KIRREL3	Associated with response to NDV during heat stress	Saelao et al. [147]
Transcriptomics	Ross 308, White Leghorn	Muscle and meat quality	JAK1, 2JAK2, TYK2	Wound healing and tissue regeneration	Zahoor et al. [156]
	Hy-Line	Liver and Muscle	HSD17B7, STARD4, ACSBG2, SCD, INSIG1,	Response to changes in energy metabolism	Wang et al. [157]
	Leghorns, Fayoumis	Lung Tissue	IL17REL	Cytokine-mediated signaling	Saelao et al. [158]
			NOX4, PRDX1, RAB7B	The phagosome maturation pathway.	
	Leghorns, Fayoumis	Bursa tissue	H3K27ac, H3K4me1	Associated with cell cycle and receptor signaling of lymphocytes.	Chanthavixay et al. [159]
	Ross 308	Blood	MYLK2, BDKRB1	Calcium signaling pathway, Response to inflammation and tissue damage	Kim et al. [160]
	Fayoumi, broilers	Thymus	FGG, IL18, IL1R2, IL13RA2	The immune response.	Monson et al. [161]
	Ross 708, Illinois	Heart	BMP10, MYH7, ANGPT2	Related to cardiovascular function	Zhang et al. [162]
	Ethiopian chickens	Heart, breast muscle, spleen	IFI27L2, F8, USP18, CEBPD	Immune response	Park et al. [163]
Proteomics	Broilers	Spleen	IL-1β, IL-6, TNF-α, IFN-α	Reveals innate immunity	Ma et al. [164]
			CTSD, PARP3, IAP3	Related to apoptosis	
			CHMP1B, TNFAIP3, PARP3, IAP3	Related to necroptosis	
	Arbor Acres	Liver	HSP90AA1, LUM, PRKAA1, LYN, ABCA1	Regulate the phagocytic ability of macrophages	Tang et al. [165]
	Ross chicks	Liver	CAT1, DLD, LDHB, ME1, PCK1, PDHA1	Carbohydrate metabolism	Kang and Shim [166]
			COX5A, COX6C, NDUFS3, UQCRC1	Energy metabolism	
			ACO2, ACAT1	Lipid metabolism	
	Taiwan country chickens	Adrenal gland	H3K27me3	Body temperature homeostasis	Zheng et al. [167]
	Taiwan country chickens	Testis	HSP90α, HSPA5, HSPA8	Attenuate the testicular injury	Wang et al. [168]
	Ross-308	Liver	MRP-126, FABP7, AGMAT, FTH1, GSTA1, TUBB, ENO1, HSP60	Response to oxidative stress	Park et al. [169]
Metabolomics	Rhode Island Red and Australorp	Egg yolk and albumen		Investigated breed and feed effects on 10 egg traits	Goto et al. [170]
	Ross 308	Breast muscle and plasma		Body energy homeostasis, growth performance, and meat quality traits	Zampiga et al. [171]
	Cobb chicks	Thigh meat		Comparing the physicochemical properties, storage stability, and metabolomic profile of thigh meat from broilers	Lee et al. [172]
	Broiler chickens	Serum		Nutrient metabolic variations	Lu et al. [173]
	Young Chickens (Chunky)	Hepatic and muscular tissue		Study was to clarify the effect of thermal conditioning at young ages on heat production and heat dissipation in chickens	Ouchi et al. [174]
	Huaixiang chickens	Serum		Lipid metabolism	Guo et al. [142]
	Arbor Acres	Bile acids		Investigating whether HS alters the composition of the bile acids pool and whether exogenous bile acids can alleviate heat stress by its characteristics described above.	Yin et al. [175]
	Arbor Acres	Serum and jejunum mucosa		Analyze some growth and antioxidative related gene expressions of jejunum mucosa	Xiong et al. [176]
	White leghorn	Kidney, liver, and breast muscle		Effects of CORT, on the metabolome of chicken kidney, liver, and breast muscle	Brown et al. [177]

**Table 5:** Comparison of genetic approaches for addressing heat stress in poultry.

Methods/Criteria	Minium Data Records	Budget (US Dollars)	Analysis Accuracy	Analysis Time	Suitability of the Area to Use the Technique	Traits	Application
Conventional	≥1000 records	≥1000	45–70%	1–2 days	Underdeveloped and developing countries	Any traits	Easy to farm animals of all sizes.
Molecular	≥50 sample/gene	≥5000	45–70%	1 week	Underdeveloped and developing countries	Any traits	Easy to farm animals of all sizes.
Genomic selection	≥300 genotyped animal records	≥100,000	>70%	At least 1 month	Developing and developed countries	Emphasis on yield and fertility traits as well as cost-reduce traits	Use in case GP and GGP farm
OMICS technology	≥5 samples	≥100,000	>90%	At least 1 month	Developed countries	Functional traits Longevity traits	Use in case GGP farm

## Data Availability

The data presented in this study are available upon reasonable request from the Network Center for Animal Breeding and Omics Research, Faculty of Agriculture, Khon Kaen University, Thailand.

## Names of Genes and Their Full Names Given in Manuscript

**Genes**	**Full names of the genes**
HSPA2	Heat shock 70 kDa protein 2
HSPH1	Heat shock 105 kDa/110 kDa protein 1,
HSP25	Heat shock protein 25
RB1CC1	RB1-inducible coiled-coil 1
BAG3	BCL2-associated athanogene 3
CITED2	Cbp/p300-interacting transactivator, with Glu/Asp-rich carboxy-terminal domain, 2
CTSE	Cathepsin E
HSPD1	Heat shock 60 kDa protein 1
ID1	Inhibitor Of DNA binding 1
HSP90B1	Heat shock protein 90 kDa beta member 1
HSP60	Heat shock protein 60
PDIA2	Protein disulfide isomerase family A member 2
HSPA5	Heat Shock Protein Family A (Hsp70) Member 5
HSF1	Heat shock factor protein 1
HSF3	Heat shock factor protein 3
HSP70	Heat shock protein 70 kDa
HSP90	Heat shock protein 90 kDa
HSP40	Heat shock protein 40 kDa
SERPINH1	Serpin family H member 1
HSP47	Heat shock protein 47
FABP2	Fatty acid binding protein 2 [(human)]
RAMP3	Receptor activity-modifying protein 3
SUGCT	Succinyl-CoA:Glutarate-CoA Transferase
TSHR	thyroid stimulating hormone receptor
GLUT-2	Glucose transporter 2
FABP1	Fatty acid binding protein 1
CD36	Cluster of differentiation 36
TRMT1L	tRNA methyltransferase 1
HS3ST5	Heparan sulfate-glucosamine 3-sulfotransferase 5
EOMES	Eomesodermin
NFAT5	Nuclear factor of activated t-cells 5
NF-κB	Nuclear factor kappa b
MRPL42	Mitochondrial ribosomal protein L42
EDN1	Endothelin 1
ACSF3	Acyl-coA synthetase family member 3
CYP4V2	Cytochrome P450 4V2
PLCB4	Phospholipase C beta 4
H1F0	H1 histone family, member 0
ACYP1	Acylphosphatase 1
JAK1	Janus kinase 1
JAK2	Janus kinase 2
TYK2	Tyrosine kinase 2
FGA	Fibrinogen alpha chain
LOXL2	Lysyl oxidase like 2
GINS1	GINS Complex Subunit 1
RRM2	Ribonucleotide reductase regulatory subunit M2
PDK	Pyruvate dehydrogenase kinase
PDK	Pyruvate dehydrogenase kinase
BVES	Blood Vessel Epicardial Substance
SMYD1	SET And MYND Domain Containing 1
IL18	Interleukin 18
PDGFRA	Platelet Derived Growth Factor Receptor Alpha
CORIN	Corin, Serine Peptidase
NRP1	Neuropilin 1
SIM2	SIM BHLH Transcription Factor 2
NALCN	Sodium Leak Channel, Non-Selective
CLPTM1L	CLPTM1 Like
APP	Amyloid Beta Precursor Protein
CRADD	CASP2 And RIPK1 Domain Containing Adaptor With Death Domain
PARK2	Parkin RBR E3 Ubiquitin Protein Ligase 2
AHR	Aryl Hydrocarbon Receptor
ESRRG	Estrogen Related Receptor Gamma
FAS	Fas Cell Surface Death Receptor
UBE4B	Ubiquitination Factor E4B
FABP1	Fatty Acid Binding Protein 1
MAP3K3	Mitogen-Activated Protein Kinase Kinase Kinase 3
SOCS2	Suppressor Of Cytokine Signaling 2
MAPKBP1	Mitogen-Activated Protein Kinase Binding Protein 1
SPON1	Spondin 1
HSP25	Heat Shock Protein 25
HSD17B1	Hydroxysteroid 17-Beta Dehydrogenase 1
APOB	Apolipoprotein B
PRDX4	Peroxiredoxin 4
SERPINH1	Serpin Family H Member 1
CIRBP	Cold Inducible RNA Binding Protein
CYP19A1	Cytochrome P450 Family 19 Subfamily A Member 1
SLC33A1	Solute Carrier Family 33 Member 1
TSHR	Thyroid Stimulating Hormone Receptor
NDUFS4	NADH:Ubiquinone Oxidoreductase Subunit S4
CAMK1d	Calcium/Calmodulin Dependent Protein Kinase ID
CCDC3	Coiled-Coil Domain Containing 3
TIRAP	TIR Domain Containing Adaptor Protein
ETS1	ETS Proto-Oncogene 1, Transcription Factor
KIRREL3	Kirre Like Nephrin Family Adhesion Molecule 3
JAK1	Janus Kinase 1
JAK2	Janus Kinase 2
TYK2	Tyrosine Kinase 2
HSD17B7	Hydroxysteroid 17-beta dehydrogenase 7
STARD4	StAR-related lipid transfer domain containing 4
ACSBG2	Acyl-CoA Synthetase Bubblegum Family Member 2
SCD	Stearoyl-CoA Desaturase
INSIG1	Insulin Induced Gene 1
ATOX1	Antioxidant 1 Copper Chaperone
SFTPA1	Surfactant Protein A1
ELK1	ETS-like 1
YY1	Yin Yang 1
ZFX	Zinc finger X-chromosomal protein
IRF3	interferon regulatory factor 3
MYLK2	Myosin Light Chain Kinase 2
BDKRB1	Bradykinin Receptor B1
FGG	Fibrinogen Gamma Chain
IL1R2	Interleukin 1 Receptor Type 2
IL13RA2	Interleukin 13 Receptor Subunit Alpha 2
BMP10	Bone Morphogenetic Protein 10
MYH7	Myosin Heavy Chain 7
PLK1	Polo Like Kinase 1
GADD45B	Growth Arrest And DNA Damage Inducible Beta
S100A8	S100 Calcium Binding Protein A8
FOS	Fos Proto-Oncogene, AP-1 Transcription Factor Subunit
CEBPD	CCAAT Enhancer Binding Protein Delta
CBFB	Core-Binding Factor Subunit Beta
SAT1	Spermidine/Spermine N1-Acetyltransferase 1
MPP1	MAGUK P55 Scaffold Protein 1
F8	Coagulation Factor VIII
NMI	N-Myc And STAT Interactor
USP18	Ubiquitin Specific Peptidase 18
CMPK2	Cytidine/Uridine Monophosphate Kinase 2
IFI27L2	Interferon Alpha Inducible Protein 27 Like 2
DHX58	DExH-Box Helicase 58
IL-1β	Interleukin 1 Beta
IL-6	Interleukin 6
TNF-α	Tumor Necrosis Factor-Alpha
IFN-α	Interferon Alpha 1
CTSD	Cathepsin D
CHMP1B	Charged Multivesicular Body Protein 1B
TNFAIP3	TNF Alpha Induced Protein 3
PARP3	Poly(ADP-Ribose) Polymerase Family Member 3
LUM	Lumican
PRKAA1	Protein Kinase AMP-Activated Catalytic Subunit Alpha 1
LYN	LYN Proto-Oncogene, Src Family Tyrosine Kinase
ABCA1	ATP Binding Cassette Subfamily A Member 1
CAT1	Catalase 1
DLD	Dihydrolipoamide Dehydrogenase
LDHB	Lactate Dehydrogenase B
ME1	Malic Enzyme 1
PCK1	Phosphoenolpyruvate Carboxykinase 1
PDHA1	Pyruvate Dehydrogenase E1 Subunit Alpha 1
COX5A	Cytochrome C Oxidase Subunit 5A
COX6C	Cytochrome C Oxidase Subunit 6C
NDUFS3	NADH:Ubiquinone Oxidoreductase Core Subunit S3
UQCRC1	Ubiquinol-Cytochrome C Reductase Core Protein 1
ACO2	Aconitase 2
ACAT1	Acetyl-CoA Acetyltransferase 1
CHGA	Chromogranin A
CHGB	Chromogranin B
HSPA5	Heat shock 70 kDa protein 5
HSPA8	Heat shock 70 kDa protein 8
HSP90AA1	Heat shock protein 90 kDa alpha, class A member 1
HSPA2	Heat shock 70 kDa protein 2
FKBP4	FK506 binding protein 4
HSP90α	Heat shock protein 90 kDa alpha
HSP70	Heat shock 70 kDa
FABP7	Fatty Acid Binding Protein 7
FTH1	Ferritin Heavy Chain 1
GSTA1	Glutathione S-Transferase Alpha 1
ENO1	Enolase 1
TUBB	Tubulin Beta Class I
